# National or International?

**Published:** 1899-01

**Authors:** G. Alden Mills


### Domestic Correspondence.

### NATIONAL OR INTERNATIONAL?

To the Editor:

Sir,—Since the session of the New York Stomatological Society in May it has been in the air that there would be a move to perfect an organization that would best represent the interests of the dental profession. At this gathering Dr. Davenport gave the central thought of the meeting, that the time had come for our profession to show what it could do for itself without the help of the trades, which, while they had been of much assistance in the hours of our need, it now seemed that we had come to an age in which we could show our independence.

We have a national body just organized, yet there seems to be evidenced by a preliminary meeting in Albany in May, and now, in the programme of the coming thirty-first anniversary of the Odontological Society of New York, there is put forth another indication that there is a decided purpose to form a national body on an advanced plan over any that has been attempted before. If this be so, then we can hope that there is a prospect of unity that has not been of late hopeful. Should a body be formed that will secure such unity it will be a matter for congratulation to the whole profession. We emphasize the *whole* profession. This may not be in the thoughts of the promoters of this movement. If all are to be benefited, then all must be represented nationally and internationally. The Odontological Society set on foot something in this direction: they brought associate interest throughout the entire civilized world, and this Society was (as can be shown by its first list of membership) a representation from among all the representative practitioners of the world. This gave a dignity that no dental society had before or since possessed. A body to represent the ability of our profession should put out an incentive for general interest and participation, so that our whole calling would have a pride in making it a body standing for the best of all which will represent us. This is what must form material for a truly professional organization that will speak for our entire profession.

What can be more certain that if this be done there will come to be a spirit of unity such as never before existed? Have we not seen too much of sectional spirit, so much so that divisions have not been promotive of the best results, to say the least? Is it not possible that out of all this petty division there may arise a structure that shall show by its fruits that there is at heart a truly ethical profession, one that shall speak to other respected professions and claim a position before the public that we could never before defend? Nothing can more certainly extradite us from the "trade spirit." All of our tendencies are on the trade lines. If we take a stand against this, professionally, we can prove that while we are dependent on the "trade" for supplies, we are not called to bury our professional honor under the *commercial idea.* That the "trade" will go on stands true; let it, on its lines. We cannot escape the fact that our calling is at heart laid on those of science applied to humanitarian principles. Commerce cannot be considered as a prime principle; it pertains only as second. It is of no concern what individuals may do, we must stand or fall on the foundation principles. Our interests are one. May we not start on this roadway, and cannot it be best secured by having an international body, with an international organ which will give expression to the best, from the best, from all parts of the civilized world? Ever, though it may be by an associate membership,

G. ALDEN MILLS.

